# A Study on the Trimming Effects on the Quality Factor of Micro-Shell Resonators Vibrating in Wineglass Modes

**DOI:** 10.3390/mi10100695

**Published:** 2019-10-12

**Authors:** Kun Lu, Xiang Xi, Dingbang Xiao, Yan Shi, Ming Zhuo, Xuezhong Wu, Yulie Wu

**Affiliations:** 1 College of Intelligence Science, National University of Defense Technology, Changsha 410073, China; luxiaofeng33@sina.com (K.L.); fordada@126.com (X.X.); shiyan_0925@sina.com (Y.S.); zhuoming592@163.com (M.Z.); xzwu@nudt.edu.cn (X.W.); ylwu@nudt.edu.cn (Y.W.); 2 Laboratory of Science and Technology on Integrated Logistics Support, National University of Defense Technology, Changsha 410073, China

**Keywords:** micro-shell resonator, quality factor, mechanical trimming, finite element method (FEM) simulation

## Abstract

Frequency trimming based on mass and stiffness modification is an important post-fabrication process for micro-shell resonators (MSRs). However, the trimming effects on the quality factor are seldom studied, although they may have great influence on the performance of the resonator. This paper presents a study on the quality factor (Q-factor) variation of trimmed micro-shell resonators (MSR). Thermoelastic damping (*Q*_TED_) and anchor loss (*Q*_anchor_) are found to be the dominant energy loss mechanisms resulting in the reduction of the overall Q-factor, according to finite element method (FEM). The effects of different trimming methods on *Q*_TED_ and *Q*_anchor_ are studied here, respectively. It is found that trimming grooves ablated in the rim of the resonator can cause a ~1–10% reduction of *Q*_TED_, and the length of trimming groove is positively related to the reduction of *Q*_TED_. The reduction of *Q*_TED_ caused by the mass adding process is mainly related to the thermal expansion coefficient and density of the additive and contact area between the resonator and additive masses. Besides, the first and second harmonic errors caused by asymmetrical trimming can cause a 10–90% reduction of *Q*_anchor_. Finally, trimming experiments were conducted on different resonators and the results were compared with FEM simulation. The work presented in this paper could help to optimize the trimming process of MSRs.

## 1. Introduction

Recently, micro shell vibrating gyroscopes (MSVGs) have shown promising performance for navigation-grade gyroscopic application, due to advantages such as a high Q-factor, structural symmetry, and immunity to external vibrations, especially with the development of micro fabrication process technologies [1,2]. As the vital component of MSVG, micro-shell resonators (MSRs) of excellent performance require a high Q-factor and matched modal frequencies between the working modes. At present, there are two different strategies to fabricate MSRs: (1) Deposition or sputtering of the resonator material (such as polysilicon, polycrystalline diamond and SiO_2_) into a spherical cavity [3,4,5,6] and (2) the plastic deformation of a device layer (such as Pyrex, metallic glass, and fused silica) using a micro glassblowing process [7,8,9]. Among these different MSRs, fused silica (FS) MSRs fabricated by micro glass-blowing show the highest Q-factor, thanks to the excellent material properties of FS, the ultra-smooth surface, and small intrinsic loss. 

The Q-factor is important to resonators because a higher Q-factor can achieve higher sensitivity, and lower noise and consumption. In order to improve the Q-factor, there have been numerous studies on energy dissipation mechanisms in these structures based on theoretical analysis and FEM simulation [10,11,12,13,14]. As a result, the structure design, fabrication process, and post treatment of the resonator has been optimized. Bulk mode resonators have been developed to achieve an ultra-high Q-factor [15,16,17]. Considering possible material defects, surface loss, and residual stress introduced in fabrication process, kinds of post-processes have been researched to improve the Q-factor of as-fabricated resonators [18,19,20].

Besides, obtaining MSRs with matched modal frequencies becomes more and more important as the sizes of the resonator decrease dramatically. As micro-machine technology has advanced, Senkal et al. have successfully fabricated MSRs with a frequency split as low as 0.1 Hz [21]. However, according to the published papers, frequency splits of most FS MSRs are still in the order of 10 Hz [3,4,5,6,7,8,9]. This is because the stability drift of MSVG is a direct function of frequency split, and post frequency tuning methods are necessary for gyroscopic application.

In general, there are two different approaches to reduce the frequency split of an imperfect resonator. The electrostatic method achieves precise frequency tuning by applying a direct current (DC) voltage on corresponding electrodes to modify the effective stiffness [22,23]. This method is often limited to tune the microelectromechanical systems (MEMS) resonator with a small initial frequency mismatch, although precise frequency tuning can be achieved. Especially for FS MSRs with a large effective stiffness, the capability of electrostatic tuning is limited with a small capacitance area and large gap. Mechanical trimming is essential for FS MSRs to achieve the permanent modification of an initial large frequency mismatch. However, mechanical trimming based on mass and stiffness perturbation may have an impact on the elastic energy variation and energy losses near the trimming position, which may reduce the Q-factor. There is a lack of specific research about the change of the Q-factor being affected by the trimming process. Therefore, it is essential to study the Q-factor of the trimmed MSR and optimize the trimming method to improve the performance of the resonator.

In our earlier study, three different MSRs were designed and fabricated. These resonators can be divided into two different kinds of structures [24,25,26,27,28]. The first one is the resonator with T-mass structures around the rim. The other structure does not have any detached mass structure. All these resonators use out-of-plane electrodes to drive and sense wineglass modes. The latest testing results showed as-fabricated MSRs can reach a frequency split less than 10 Hz in the wineglass modes without any post-process [24]. Although a systematic trimming process was successfully applied to reduce the frequency split of MSRs [25,26], the impact on the Q-factor has not been specifically researched. This paper intends to study the change of overall the Q-factor of the trimmed MSRs by FEM simulation and experiments.

The paper is organized as follows. The basic structures of MSRs and trimming process are briefly introduced in Section 2. The change of the Q-factor in trimmed resonators is qualitatively analyzed based on the energy loss mechanism. Then, FEM simulation is utilized to calculate the change of *Q*_TED_ and *Q*_anchor_ in trimmed resonators separately in Section 3. Finally, Section 4 presents the experimental results of different resonators and discusses the differences between the experimental and simulation results.

## 2. Problem Characterization

### 2.1. Structure Description

**Figure 1:**
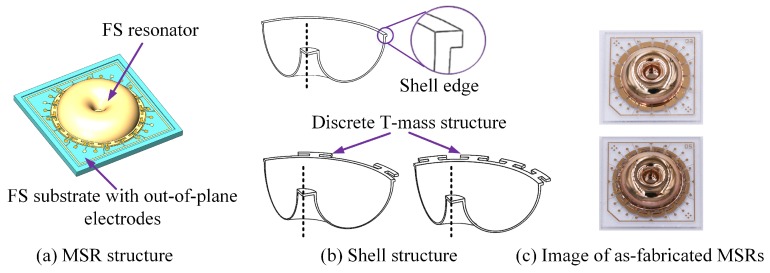
(**a**) Structure of micro-shell resonators (MSRs), containing the shell resonator and substrate with an out-of-plane electrode; (**b**) Different shell structures of the resonators; (**c**) Image of as-fabricated MSRs.

The structure of the MSR consists of the shell resonator and out-of-plane electrode, as shown in Figure 1a. The shell structure is attached to the FS substrate at the central anchor. Distinguished by edge structures, there are three different MSRs in our research, shown in Figure 1b. These different discrete mass structures are designed to adjust the dynamic parameters of the resonator by regulating the effective stiffness and mass. Figure 1c shows an image of the as-fabricated MSRs.

**Figure 2:**
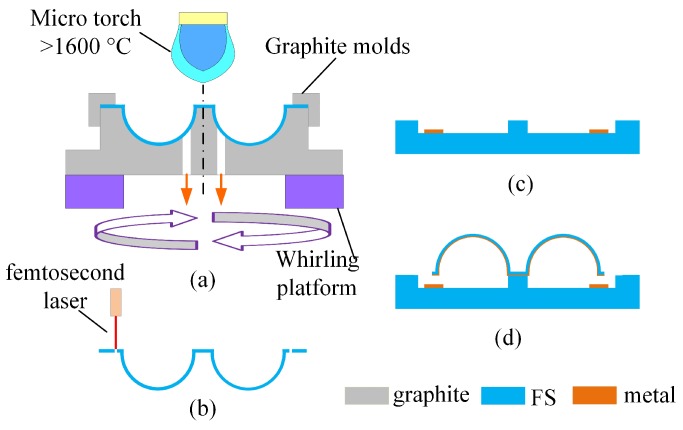
Schematic diagram of fabrication process steps for MSRs. (**a**) The FS is reshaped to a shell structure; (**b**) Shell edges are released by femtosecond laser and the interior surface of the resonator is then metalized; (**c**) The FS substrate is patterned with electrodes; (**d**) The metalized resonator is attached to the substrate.

These MSRs are fabricated using micro blow torching with a whirling platform and femtosecond ablation process [27,28]. The FS is firstly reshaped to a shell structure, whirling at a high rotational speed (Figure 2a). Different shell edges are released by a femtosecond laser and the interior surface of the resonator is then metalized (Figure 2b). The FS substrate is patterned with electrodes (Figure 2c). Finally, the metalized resonator is attached to the substrate, creating capacitive gaps between the metalized resonator and the electrodes (Figure 2d). 

**Figure 3:**
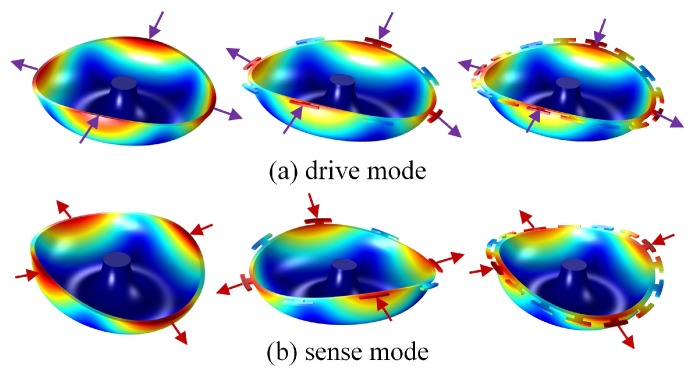
*n* = 2 wineglass modes of MSRs. The nodes and anti-nodes are separated by 45°. (**a**) Drive modes of the MSRs; (**b**) Sense modes of the MSRs.

These MSRs operate in wineglass four-node modes. Figure 3 shows the wineglass mode contours in the resonators. For a perfectly symmetrical MSR used as a yaw gyroscope, the resonant frequency of the first and second wineglass modes must be well matched. Besides, the nodes and anti-nodes of the modes should be aligned with the drive and sense electrodes. According to the latest testing results, the Q-factor of the three different resonators is 100–450 k in a 1 × 10^−3^ pa vacuum condition. However, the frequency split of the resonators is 3–30 Hz in *n* = 2 wineglass modes, which need to be trimmed for gyroscopic application [29].

### 2.2. Trimming of FS MSRs

**Figure 4:**
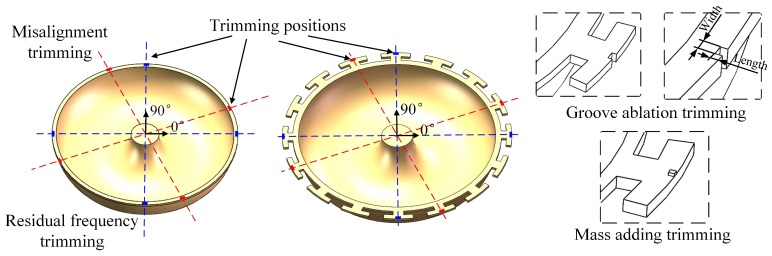
Different trimming methods on the edge of resonators.

Limited by fabrication precision, as-fabricated MSRs have frequency splits and modal cross coupling. Mass and stiffness modification can be used to reduce the frequency mismatch. Groove ablation has been demonstrated to be useful for frequency trimming in MSRs. Besides, the material added in the discrete mass structures can achieve frequency trimming by modifying the mass condition, which will discuss in subsequent section. Actually, there are two different positions for mechanical trimming, as shown in Figure 4. Grooves ablated in the shell structure (or edge between the discrete mass structure) achieve frequency trimming mainly by decreasing stiffness in corresponding direction. On the other hand, effective mass can be modified by ablating grooves or adding mass point in discrete T-mass structures.

Let the coordinate of each trimming mass be (*m_i_*, *φ_i_*) and each trimming stiffness *k_j_* be (*k_j_*, *ϕ_j_*), thus the mode orientation angle of high-frequency *ϕ*_1_ and frequency split (Δ*ω*) can be calculated by [30,31]:

(1) $$\begin{array}{l} \varphi_{1}=\arctan\left\{\frac{(\sigma_{s}+\lambda_{m}\sum_{i}m_{i}\sin4\phi_{i}}{(\sigma_{c}+\lambda_{m}\sum_{i}m_{i}\cos4\phi_{i}}\right\}\\ \Delta \omega =\sqrt{{\left(\sigma_{c}+\lambda_{m}\sum_{i}m_{i}\cos4\phi_{i}\right)}^{2}+{\left(\sigma_{s}+\lambda_{m}\sum_{i}m_{i}\sin4\phi_{i}\right)}^{2}} \end{array}$$

(2) $$\begin{array}{l} \varphi_{1}=\arctan\left\{\frac{(\sigma_{s}+\lambda_{k}\sum_{j}k_{j}\sin4\phi_{j}}{(\sigma_{c}+\lambda_{k}\sum_{j}k_{j}\cos4\phi_{j}}\right\}\\ \Delta \omega =\sqrt{{\left(\sigma_{c}+\lambda_{k}\sum_{j}k_{j}\cos4\phi_{j}\right)}^{2}+{\left(\sigma_{s}+\lambda_{j}\sum_{j}k_{j}\sin4\phi_{j}\right)}^{2}} \end{array}$$

where *λ_m_* and *λ_k_* are constant, determined by the geometry and material of the resonator. The corresponding low-frequency axis for the coupling eigenmode is *ϕ*_2_, *ϕ*_2_ = *ϕ*_1_ − 45°. The imbalance parameters, *σ_s_* and *σ_c_,* can be calculated as follows:

(3) $$\begin{array}{l} \sigma_{c}=\left(\omega_{1}-\omega_{2}\right)\cos4\varphi \\ \sigma_{s}=\left(\omega_{1}-\omega_{2}\right)\sin4\varphi \end{array}$$

For an imperfect MSR, the frequency split can be eliminated by reducing stiffness in the 22.5° and 112.5° directions (near the high-frequency axis). Also, it can be eliminated by reducing mass in the 0° and 90° directions (near the low-frequency axis) or increasing mass in the 45° and 112.5° directions (near the high-frequency axis).

### 2.3. Quality Factor of the Trimmed Resonator

**Figure 5:**
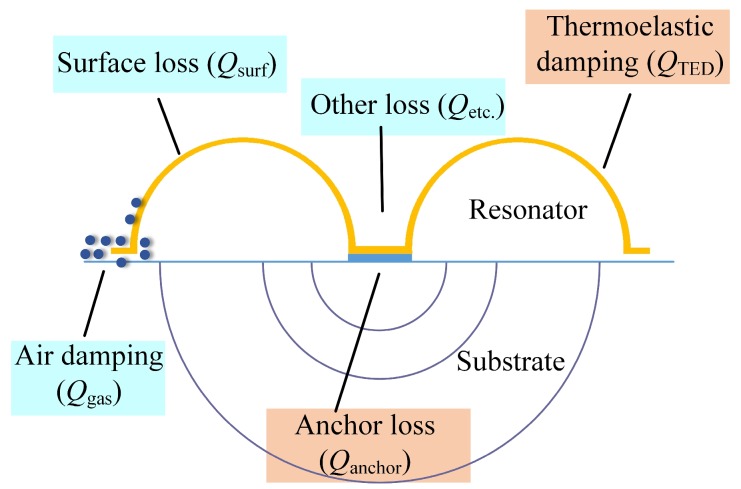
Major dissipation mechanism in trimmed MSRs: Air damping loss, anchor loss, surface loss, thermoelastic damping loss and additional loss mechanism.

The overall Q-factor of the trimmed MSR can be calculated from the contribution of different dissipation mechanisms, such as air damping loss, anchor loss, surface loss, thermoelastic damping loss, and additional loss mechanism, as shown in Figure 5. The Q-factor from these energy dissipations can be added as inverses, shown in Equation (4). The relationship indicates that the overall Q-factor is mainly limited by the loss mechanism with the lowest *Q*-factor. In order to confirm the main factors causing the reduction of the Q-factor, changes of different energy dissipation during the trimming process were analyzed.

(4) $$\frac{1}{Q}=\frac{1}{Q_{gas}}+\frac{1}{Q_{\mathrm{anchor}}}+\frac{1}{Q_{\mathrm{surf}}}+\frac{1}{Q_{\mathrm{TED}}}+\frac{1}{Q_{\mathrm{etc}.}}$$

- Air damping is the most dominant factor when the resonator works at atmospheric conditions, including squeeze film damping and slide film damping. It can be eliminated by operating the resonator in a high vacuum condition. As for MSRs, the most concerned air damping is caused by the vibration of flat rim. Because the trimming process would not increase the area of the resonator rim, the *Q*_gas_ could hardly change. Hence, the groove trimming process cannot increase air damping.
- Surface loss is often believed to be caused by defects, roughness, and other imperfections on the surface of the resonator [32]. At present, there is no analytical formula to calculate *Q*_surf_, and modeling concerning *Q*_surf_ is still controversial. Besides, surface roughness is generally thought to be a main factor of the *Q*_surf_. In our research, trimming grooves are ablated by a femtosecond laser, which uses the same process as the releasing of the shell structure. Thus, the effects of trimming on *Q*_surf_ are minimized through the femtosecond laser to achieve a smooth surface quality of the trimming grooves.
- Thermoelastic damping loss is caused by the interaction between elastic strain and thermal effects. When the MSR vibrates in the wineglass mode, some regions are under compression while others are under extension. In those conditions, irreversible heat flow occurs from the warmer parts of the structure to the cooler parts, and this heat flow is associated with energy loss [33]. Trimming grooves may have an impact on the stress distribution and elastic energy variation, resulting in more damping loss. Besides, the mass adding trimming method may cause a reduction of *Q*_TED_ because it could result in more heat generation and dissipation between the resonator and the added masses.
- Anchor losses occur when the vibration of the MSR and its supporting anchors excites acoustic waves propagating in the substrate [34]. These waves are radiated away from the resonator, resulting in a loss of mechanical energy. Trimming grooves may affect the coupling of the resonant mode to the substrate and result in more anchor loss.

## 3. FEM Simulation of Quality Factor

While Equation (4) can help to understand the mechanism of the overall Q-factor, it is hard to calculate the Q-factor of the trimmed resonator because we cannot get an accurate expression about energy dissipation. However, FEM simulation provides the possibility to solve this problem. In this section, COMSOL Multiphysics (Editon 5.3, Comsol Inc, Burlington, MA, USA) is used to numerically calculate the Q-factor of different dissipation mechanisms. Considering the possible change caused by trimming grooves on the resonator, *Q*_TED_ and *Q*_anchor_ are calculated.

### 3.1. FEM Simulation of Q*_TED_* of Trimmed MSRs

Thermoelastic dissipation is caused by an interaction between thermal fluctuation and mechanical vibration. In order to calculate *Q*_TED_, a thermoelastic physical field was introduced and the coupled thermo-mechanical eigenvalue was solved in a simulation. For a resonator, the Q-factor can be calculated using following equation:

(5) Qi=|Re(ωi)2Im(ωi)|

where *Q_i_* and *ω_i_* are the Q-factor and eigenvalue of the *i*th mode. *Q*_TED_ can be calculated by applying COMSOL Multiphysics to solve the coupled thermo-mechanical eigenvalue.

#### 3.1.1. Effect of Removing Masses on *Q*_TED_

**Figure 6:**
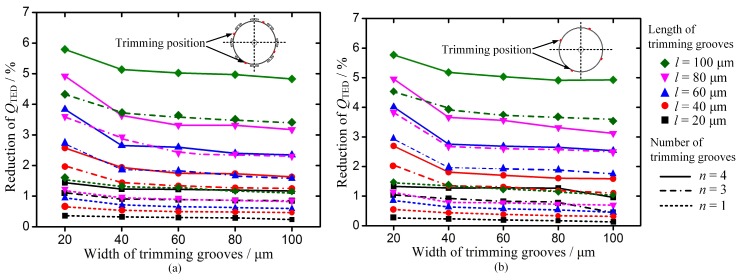
Effect of the dimensions and number of trimming grooves ablated in the rim of the resonator on reduction of *Q*_TED_ in low-frequency mode. (**a**) Reduction of *Q*_TED_ when grooves ablated between T-mass. (**b**) Reduction of *Q*_TED_ when grooves are ablated in the rim of the MSR without mass structures.

For a symmetrical MSR, *Q*_TED_ is calculated to be about 26 million (*Q*_0_). The simulation results indicate that grooves ablated in the edge of discrete mass structures only cause a slight decrease of *Q*_TED_. As for these structures, the reduction of *Q*_TED_ is less than 0.05% as groove sizes increase. However, grooves ablated between the mass structure and grooves ablated in the edge of resonator without mass structures cause a significant reduction of *Q*_TED_. This kind of mass removal from the rim could cause more stiffness reduction in the corresponding direction to balance the wineglass modes. For these trimmed resonators, *Q*_TED_ in the low-frequency mode reduces much more than the other one, because the trimming grooves are located at the nodes of the low-frequency mode. The vibration along the trimming grooves causes more energy dissipation and more reduction in *Q*_TED_. Figure 6 shows the effects on the reduction of *Q*_TED_ in low-frequency mode when the trimming grooves ablated in these positions.

It can be found that *Q*_TED_ decreases obviously as the length of the grooves increases, and it decreases as the width of grooves decreases. The reduction of *Q*_TED_ in these resonators is sensitive to the length variation of the trimming groove. Besides, the reduction of *Q*_TED_ is in direct proportion to the number of trimming grooves. Therefore, in order to minimize its effect on *Q*_TED_, grooves could be fixed on a short length. Additionally, increasing the width of the groove to modify the stiffness can be applied in the practical trimming process.

**Figure 7:**
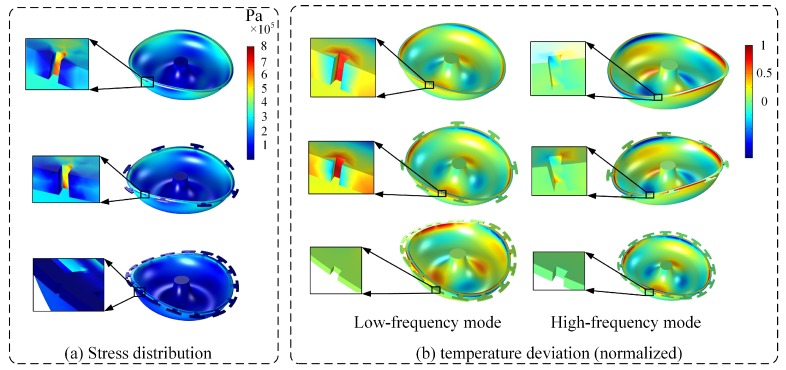
Stress distribution and temperature deviation of MSR when grooves ablated in different position. (**a**) Stress distribution in trimmed MSRs; (**b**) Temperature deviation in trimmed MSRs.

In order to understand the reduction mechanism of *Q*_TED_, the distribution of stress and temperature deviation was simulated when grooves were ablated in different positions, as shown in Figure 7. Grooves ablated in the edge of mass structure have little impact on the concentration of stress and temperature departure, hardly affecting *Q*_TED_. However, trimming grooves ablated between the mass structures cause a concentration of stress and temperature departure, which can result in more dissipation in *Q*_TED_. Besides, grooves ablated in these positions cause more temperature deviation over a larger area in the low-frequency mode than that in high-frequency mode. Thus, the reduction of *Q*_TED_ in the low-frequency mode is much more than that in the high-frequency mode after groove trimming in the edge of MSR without mass structures.

#### 3.1.2. Effect of Added Masses on *Q*_TED_

As for MSRs with mass structures, mode frequencies can be modified by adding masses in these discrete structures, aligned with the high-frequency axis. The added material could cause a change in the thermo-mechanical behavior of the resonator, because it may have different properties from the fused silica. These properties include *E* (Young modulus), *C*_P_ (constant pressure heat capacity), *ρ* (density), *υ* (Poisson’s ratio), *α* (coefficient of thermal expansion), and *k* (coefficient of thermal conductivity). 

**Figure 8:**
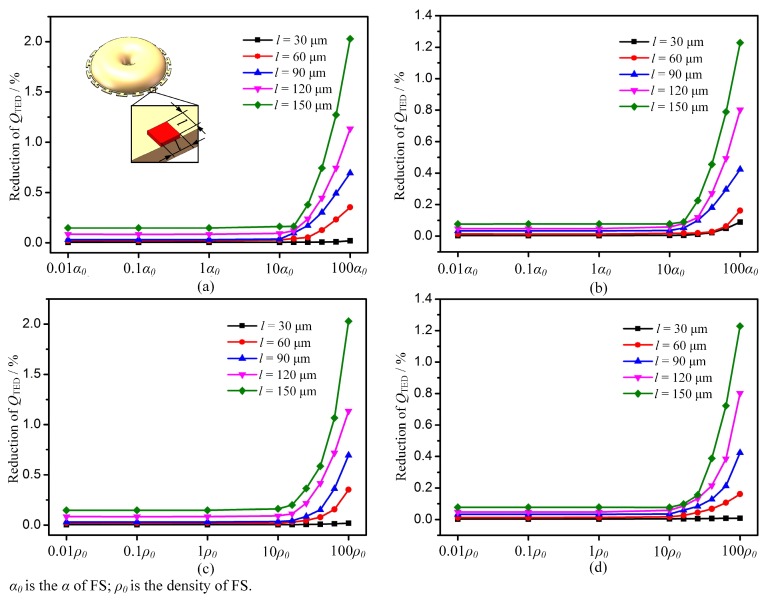
Effect of α (coefficient of thermal expansion) and ρ (density) of added mass on change in *Q*_TED_. (**a**,**c**) Reduction of *Q*_TED_ in the low-frequency mode. (**b**,**d**) Reduction of *Q*_TED_ in the high-frequency mode.

The change of *Q*_TED_ was simulated versus the change in added material properties when adding masses in MSRs with sixteen T-mass structures. In each simulation, one property of added mass was changed while the others were confined at values of the fused silica. The total weight of the added mass was fixed when changing the contact area for the sake of minimizing the impact of the mass weight. The simulation results show that *E*, *ρ*, *υ*, *α*, and *k* have little impact on the reduction of *Q*_TED_. The greatest impact factors are the *α* and *ρ* of the added mass, as shown in Figure 8.

Another important factor is the contact area between the resonator and the added masses. As the contact area increases, *Q*_TED_ decreases sharply. This is because larger contact regions can cause more heat generation and dissipation between resonator and added masses. Hence, reducing the contact area is beneficial to improving *Q*_TED_ during the trimming process. Considering that the density of the added material has little influence on the *Q*_TED_, materials with high densities can be used to decrease the contact area.

### 3.2. FEM Simulation of Q*_anchor_* of Trimmed MSRs

**Figure 9:**
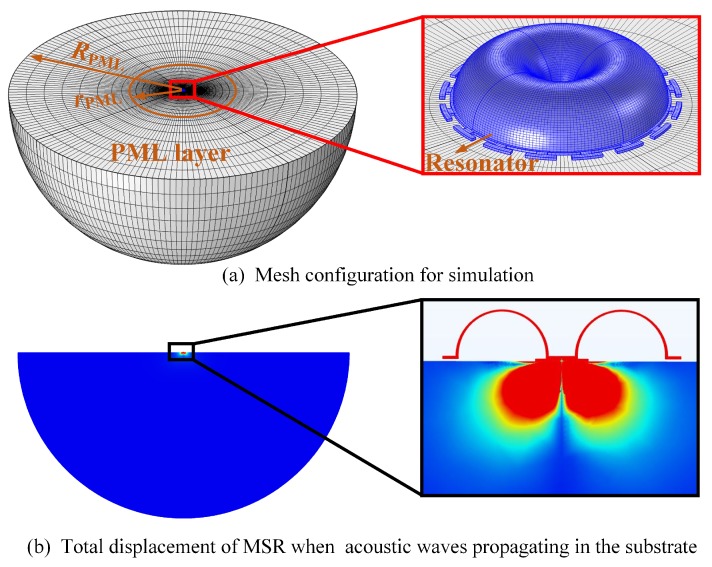
(**a**) One of mesh configuration for *Q*_anchor_ simulation in a MSR with sixteen T-masses. (**b**) Total displacement of the resonator when acoustic waves propagate in the substrate.

Anchor losses occur when the vibration of the resonator and its supporting anchors excites acoustic waves propagating in the substrate. Because the outgoing waves should be considered in an infinite area, a perfect match layer (PML) was introduced as an absorbing layer [13]. A model including the MSR, finite substrate, and PML, absorbing the waves propagating in the substrate, was built, as shown in Figure 9. The structure of the finite substrate and PML were spherical, and all the structures in the model are made of fused silica. The thickness and mesh quality of the different layers in the model are critical to the accuracy of the calculated *Q*_anchor_ value. The thickness of PML (*R*_PML_ − *r*_PML_) was confined to the wavelength of the propagating wave. In order to improve the calculation accuracy and decrease solving time, mesh density varies from intensive in the regions close to the substrate to sparse in the region close to the PML outer boundary.

A simulation of modal analysis was implemented and Equation (6) was used to calculate *Q*_anchor_. For a symmetrical resonator, *Q*_anchor_ was calculated to be about 60 billion (*Q*_0_). *Q*_anchor_ was then calculated for a MSR with a different trimming process. The FEM results show that the trimming process has little impact on the Q-factor when grooves or added masses are symmetrically distributed around the center of resonator. For example, when 4, 8, or 16 trimming grooves or added masses are symmetrically distributed around the resonator, the reduction of *Q*_anchor_ is less than 0.5%. 

**Figure 10:**
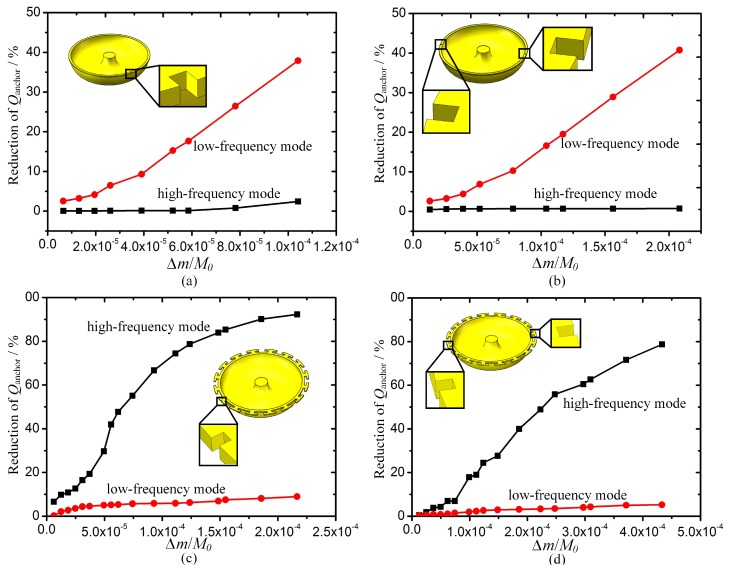
Effect of asymmetric trimming grooves on reduction of *Q*_anchor_. (**a**) Reduction of *Q*_anchor_ when a groove ablated in MSR without mass structures; (**b**) Reduction of *Q*_anchor_ when two grooves ablated in MSR without mass structures;(**c**) Reduction of *Q*_anchor_ when a groove ablated in MSR with sixteen T-mass structures;(**d**) Reduction of *Q*_anchor_ when two grooves ablated in MSR with sixteen T-mass structures.

However, there might be an asymmetrical error caused by the precision and location error of the laser trimming. Specifically, there are mainly first and second harmonic errors introduced in the four-points trimming method for wineglass modes. Figure 10 shows the reduction of *Q*_anchor_ in wineglass modes when the first and second harmonic errors are introduced. In the simulation, one and two symmetrically distributed grooves present the two different harmonic errors separately. According to the FEM results, asymmetrical trimming has a great influence on the reduction of *Q*_anchor_ along the trimming grooves. However, reduction of *Q*_anchor_ deviating from the trimming grooves is within 10%. When there is an asymmetric groove, the MSR vibrates in the mode along the groove, while the other mode is 45° away from the trimming groove. Thus, the wineglass mode along asymmetric groove is affected much more than the other one.

A similar rule can be found when asymmetrically distributed added masses are used in the trimming process. According to Equation (5), the loss mechanism with the lowest Q-factor determines the overall Q-factor of the MSR. Although the reduction percentage of *Q*_anchor_ is much more than that of *Q*_TED_, the change of *Q*_TED_ still has a larger impact on the overall Q-factor because the initial *Q*_anchor_ is more than 1000 times that of *Q*_TED_.

## 4. Experiments and Discussion

**Figure 11:**
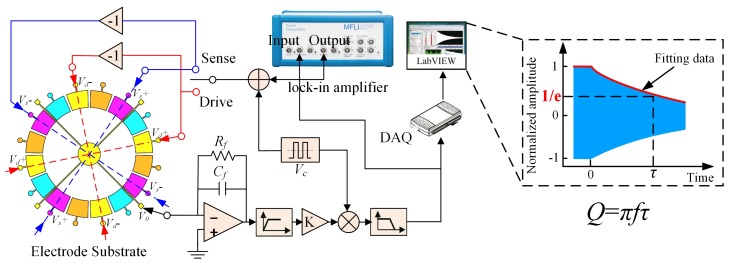
Block diagram of the experimental setup for Q-factor measuring in wineglass modes.

The FEM simulation indicates that the trimming process has a significant influence on the overall Q-factor of MSRs. In this section, experiments were implemented to validate the FEM simulation in Section 3. Three different kinds of MSRs, which had initial frequency splits of 10–50 Hz, were used in the experiments. All resonators were operated under high-vacuum conditions (0.01Pa) to minimize the effect of air damping. The Q-factors of these resonators were calculated by measuring the ring-down time (*τ*) with a lock-in amplifier, as shown in Figure 11. The resonators were driven in wineglass modes using a lock-in amplifier to sustain a certain vibration amplitude. Then, the driving voltage was turned off, and the ring-down time could be tested when the vibration amplitude reached 1/e of the original amplitude.

**Figure 12:**
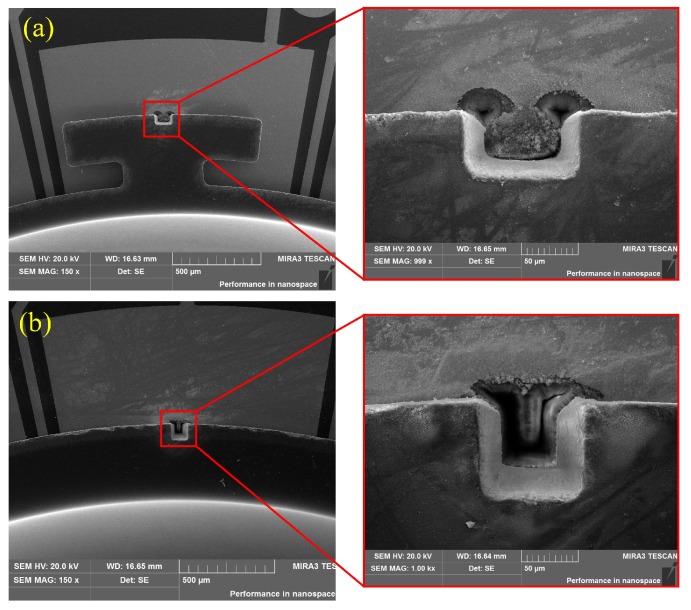
SEM of trimming grooves ablated in different position. (**a**) Groove ablated in the edge of T-mass structure. (**b**) Groove ablated in the rim between T-masses.

Three different sets of comparative experiments were conducted. The first group was designed to verify the effects of trimming grooves on the Q-factor of different MSR structures. The groove trimming processes mentioned in Section 2 were applied to the MSRs to eliminate frequency splits below 0.5 Hz, which is necessary for the operating of gyroscopes. Detailed trimming methods can be found in [26]. Figure 12 shows the feature of grooves ablated by femtosecond laser. The Q-factor along drive and sense axes was separately measured. The modal contour close to the initial high-frequency axis was confined as the drive mode. The results presented in Table 1 indicate that the Q-factor of MSRs with sixteen T-masses hardly change after groove trimming. However, there is a significant reduction in the Q-factor of MSRs without mass structures as well as MSRs with eight T-masses. Besides, it is noted that the Q-factor reduces more in drive modes because grooves ablated in the rim are close to the high-frequency axis.

**Figure 13:**
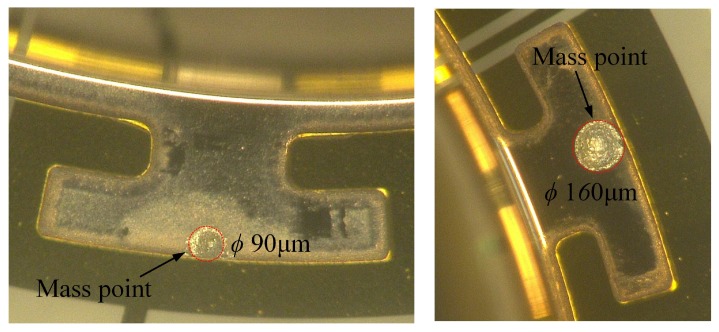
Feature of added mass point in the edge of T-masses. A kind of conducting resin (DAD 51) is used as the added material.

The second group was designed to verify the impact of adding mass trimming on the Q-factor of MSRs with discrete mass structures. MSRs with sixteen T-masses were used in the experiments. A kind of adhesive (conducting resin DAD-51) was used as the added material for frequency trimming, as shown in Figure 13. The adhesive was added on the edge of the T-mass structure and solidified at 150 degrees Celsius for 30 minutes. The results presented in Table 2 show that the reduction percentage of Q-factor is sensitive to the contact area between the adhesive and resonator. Besides, the experimental results show a much larger reduction of the Q-factor than *Q*_TED_ simulated in Section 3. This is because the surface loss and contact stress between the resonator and added mass are not considered in the simulation.

The last set of contrast experiments were conducted to study the impact of asymmetrical trimming on the overall Q-factor of MSRs. Groove trimming on MSRs with sixteen T-masses was conducted in order to minimize the influence of *Q*_TED_. First, harmonic errors were introduced in samples No.13–No.15, and second harmonic errors were introduced in samples No.16–No.18. The experiment results presented in Table 3 indicate that asymmetric trimming has little impact on the change of Q-factor in wineglass modes. There are two reasons for this result. Firstly, there were some first and second harmonic errors in the resonator before it was trimmed. The asymmetric trimming just caused a relative smaller modification for these errors. Besides, the main limitation of the overall Q-factor is *Q*_TED_ at present. The reduction of *Q*_anchor_ caused by asymmetric trimming has a small influence on the overall Q-factor.

## 5. Conclusions

The change of *Q*_TED_ and *Q*_anchor_ has been calculated by FEM simulation in trimmed MSRs. The simulation results indicate that groove trimming in the rim of the resonator causes a significant reduction of *Q*_TED_, and the length of the trimming grooves is positively related to the reduction of *Q*_TED_. As for MSRs with discrete mass structures, adding mass trimming only slightly reduces *Q*_TED_ according to simulation results. First and second harmonic errors introduced by asymmetric trimming can result in a significant reduction of *Q*_anchor_. The reduction percentage is positive to the weight of imbalance mass.

Furthermore, three different contrast experiments were conducted to verify the FEM simulation. The experiment results show that the reduction of Q-factor in trimmed MSRs without mass structures, as well as MSRs with eight T-masses, is coincident with the change of *Q*_TED_ in the FEM simulation. However, adding mass trimming in MSRs with discrete structures shows a much larger reduction of Q-factor than *Q*_TED_ in the simulation because of the extra surface loss introduced by the adhesive. Besides, asymmetrical trimming has little impact on the overall Q-factor of the MSR, though it can cause a significant reduction in *Q*_anchor_.

The investigation on change of trimmed MSRs presented in this paper can be helpful for optimizing mechanical trimming process for MSRs.

## Figures and Tables

**Table 1:** Q-factor change of different MSRs after trimmed by groove ablation.

Type of MSRs		Initial Δ*f*/Hz	Original Q		Q after Trimming		Reduction of Q	
			*Q*_drive_	*Q*_sense_	*Q*_drive_	*Q*_sense_	Drive Mode	Sense Mode
MSR with 16 T-masses	No.1	13.3	168.5 k	179.3 k	165.5 k	175.1 k	1.78%	2.34%
	No.2	15.8	136.8 k	125.9 k	133.6 k	121.9 k	2.34%	3.18%
	No.3	20.7	156.9 k	150.2 k	153.7 k	145.5 k	2.04%	3.13%
MSR with 8 T-masses	No.4	20.82	129.4 k	134.3 k	110.7 k	118.2 k	14.45%	11.99%
	No.5	35.5	135.5 k	145.1 k	113.8 k	138.9 k	16.01%	4.27%
	No.6	46.07	122.2 k	119.8 k	101.2 k	109.7 k	17.18%	8.43%
MSR without T-masses	No.7	15.4	145.6 k	147.2 k	125.3 k	138.2 k	13.94%	6.11%
	No.8	19.7	149.7 k	156.6 k	128.1 k	141.8 k	14.43%	9.45%
	No.9	22.5	161.3 k	143.8 k	139.7 k	129.9 k	13.39%	9.66%

**Table 2:** Q-factor change of MSRs after trimmed by mass adding process.

Samples	Initial Δ*f*/Hz	Diameter of Mass/μm	Original Q		Q after Trimming		Reduction of Q	
			*Q*_drive_	*Q*_sense_	*Q*_drive_	*Q*_sense_	Drive Mode	Sense Mode
No.10	20.7	*~ ϕ* 80	157.1 k	168.4 k	139.8 k	155.6 k	11.01%	7.60%
No.11	23.5	*~ ϕ* 120	179.6 k	171.5 k	150.9 k	158.1 k	15.98%	7.81%
No.12	18.2	*~ ϕ* 165	149.8 k	155.7 k	120.6 k	138.3 k	19.49%	11.2%

**Table 3:** Comparison of Q-factor change when first and second harmonic errors introduced in trimming process.

Samples	Initial Δ*f*/Hz	Original Q		Q after Trimming		Reduction of Q	
		*Q*_drive_	*Q*_sense_	*Q*_drive_	*Q*_sense_	Drive Mode	Sense Mode
No.13	12.8	175.9 k	161.2 k	171.1 k	157.6 k	2.73%	2.23%
No.14	18.7	136.4 k	150.1 k	134.3 k	147.1 k	1.54%	2.00%
No.15	21.6	167.3 k	175.7 k	164.5 k	170.2 k	1.67%	3.13%
No.16	13.9	146.8 k	161.9 k	144.1 k	157.6 k	1.83%	2.66%
No.17	16.4	131.9 k	129.1 k	128.2 k	124.3 k	2.81%	3.72%
No.18	23.8	153.8 k	162.7 k	150.9 k	157.1 k	1.89%	3.44%
